# Identification of an age-dependent biomarker signature in children and adolescents with autism spectrum disorders

**DOI:** 10.1186/2040-2392-4-27

**Published:** 2013-08-06

**Authors:** Jordan M Ramsey, Paul C Guest, Jantine AC Broek, Jeffrey C Glennon, Nanda Rommelse, Barbara Franke, Hassan Rahmoune, Jan K Buitelaar, Sabine Bahn

**Affiliations:** 1 Department of Chemical Engineering and Biotechnology, University of Cambridge, Tennis Court Road, Cambridge CB2 1QT, UK; 2 Department of Cognitive Neuroscience, Donders Institute of Brain, Cognition and Behaviour, Radboud University Nijmegen Medical Centre, Geert Grooteplein-Noord 21, Nijmegen 6525 EZ, The Netherlands; 3 Department of Psychiatry, Donders Institute of Brain, Cognition and Behaviour, Radboud University Nijmegen Medical Centre, Geert Grooteplein-Noord 21, Nijmegen 6525 EZ, The Netherlands; 4 Karakter Child and Adolescent Psychiatry University Centre, Geert Grooteplein-Zuid 16, Nijmegen 6525 GA, The Netherlands; 5 Department of Genetics, Donders Institute of Brain, Cognition and Behavior, Radboud University Nijmegen Medical Center, PO Box 9101, Nijmegen 6500 HB, The Netherlands; 6 Department of Neuroscience, Erasmus Medical Centre, 's-Gravendijkwal 230, Rotterdam 3015 CE, The Netherlands

**Keywords:** Autism, Age, Biomarkers, Molecular profiling, Inflammation, Metabolism

## Abstract

**Background:**

Autism spectrum disorders (ASDs) are neurodevelopmental conditions with symptoms manifesting before the age of 3, generally persisting throughout life and affecting social development and communication. Here, we have investigated changes in protein biomarkers in blood during childhood and adolescent development.

**Methods:**

We carried out a multiplex immunoassay profiling analysis of serum samples from 37 individuals with a diagnosis of ASD and their matched, non-affected siblings, aged between 4 and 18 years, to identify molecular pathways affected over the course of ASDs.

**Results:**

This analysis revealed age-dependent differences in the levels of 12 proteins involved in inflammation, growth and hormonal signaling.

**Conclusions:**

These deviations in age-related molecular trajectories provide further insight into the progression and pathophysiology of the disorder and, if replicated, may contribute to better classification of ASD individuals, as well as to improved treatment and prognosis. The results also underline the importance of stratifying and analyzing samples by age, especially in ASD and potentially other developmental disorders.

## Background

Autism spectrum disorders (ASDs) are a clinically and biologically heterogeneous group of neurodevelopmental conditions characterized by a triad of core features: social and communication impairments and restricted repetitive behavior. The clinical manifestations of ASD have been shown to change over development. Cross-sectional and longitudinal research indicates that the severity of the core features and maladaptive behaviors of ASD among adolescents and adults tend to abate with age [1-4]. A cross-sectional study showed improved gaze behavior and social functioning of ASD subjects between adolescence and adulthood, with the suggestion that increased mirror neuron system activity may contribute to these effects [5].

In addition to the clinical manifestations, there is accumulating evidence that individuals with ASD have significant differences in brain development compared to controls. The results of several studies that were reviewed in [6] have shown there is reduced functional activation in multiple brain areas of 2-to 4-year-old children during socio-emotional, cognitive and attention tasks. Also, studies have shown age-dependent changes in cortical development [7] in brain regions involved in social-cognitive and motor function [8], language [9], and symptom severity [10]. Taken together, the findings indicate that neurobiological alterations that occur during the first years of life may underlie the neuroanatomical, functional and behavioral aspects of ASD. Therefore, identification of biomarkers associated with these alterations may provide further insights into the disease etiology.

Thus far, there have been only a small number of studies that have attempted to identify molecular changes in ASD that occur at different ages. One study found age-dependent gene expression changes in prefrontal cortex using whole-genome analysis of mRNA levels in post-mortem brains of ASD subjects [11]. Most of the molecular profiling studies have investigated age-related changes in ASD subjects in the levels of growth factors such as brain-derived neurotrophic factor (BDNF). In ASD cases, the levels of BDNF were found to be significantly lower in 0- to 9-year-old children compared to those aged greater than 10 years, while no age-related differences in BDNF levels were found for non-ASD controls [12]. This suggested that there may be a delayed increase of BDNF with development. The ^1^H nuclear magnetic resonance (NMR) analyses found lower frontal lobe ratios of N-acetylaspartate/creatine, which was correlated with age in ASD children [13]. This could reflect increased mitochondrial metabolism and may be related to symptoms of obsessional behavior and decreased social function of the patients.

Most previous molecular profiling studies of ASD have been performed using specific age groups, which precludes identification of changes that occur at different stages of development. Here we have attempted to gain further insight into age-related molecular trajectories in ASD by multiplex immunoassay profiling of 208 analytes in serum from patients and sibling controls, following partitioning into three age groups (4 to 9, 9 to 13 and 13 to 18 years). This platform has the advantage of being capable of screening multiple molecules simultaneously in biological samples and has been used previously to identify serum or plasma biomarkers in several areas of medicine, including neuropsychiatric conditions such as schizophrenia, bipolar disorder, major depressive disorder and Asperger syndrome [14-16].

## Methods

### Subjects

Subjects were recruited from Karakter Child and Adolescent Psychiatry and the Radboud University Nijmegen Medical Center in Nijmegen, The Netherlands. The subjects included 37 ASD subjects (age = 10.8 ± 3.5 years; body mass index (BMI) = 18.0 ± 3.7 kg/m^2^) and 37 controls (age = 10.5 ± 3.2 years; BMI = 17.6 ± 3.0 kg/m^2^). The Commissie Mensgebonden Onderzoek (CMO) regio Arnhem Nijmegen ethical committee approved the study protocols, informed written consent was given by the parents of all participants, and studies were conducted according to the Declaration of Helsinki. Clinical diagnosis of ASD was conferred by board certified child psychiatrists based on developmental history and psychiatric interview and observation and according to accepted international criteria (APA, DSM-IV-TR). Diagnosis of ASD was confirmed by a structured developmental interview with the parents (ADI-R) [17]. Subjects with a diagnosis of autistic disorder (AD) or pervasive developmental disorder-not otherwise specified (PDD-NOS) were included in the study. The Wechsler Abbreviated Scale of Intelligence was administered to all participants to measure intelligence quotient, and age-appropriate Autism Spectrum Quotient (AQ) questionnaire scores were recorded for all ASD and control individuals. All diagnoses and clinical tests were performed by psychiatrists under Good Clinical Practice compliance to minimize variability. Unaffected control subjects were siblings recruited from the same families and had comparable age, gender and body mass index (BMI) to the respective patient populations.

### Samples

Blood samples were collected from all ASD individuals and controls into S-Monovette 7.5 mL serum tubes (Sarstedt, Numbrecht, Germany). Serum was prepared using standard protocols by leaving samples at room temperature for 2 hours to allow clotting, followed by centrifugation at 4,000 × g for 5 minutes to remove clotted cells and other particulate material. The resulting supernatants were stored at −80°C in LoBind Eppendorf tubes (Hamburg, Germany). The study protocols, processing of clinical samples and execution of test methods were carried out in compliance with the Standards for Reporting of Diagnostic Accuracy (STARD) initiative [18].

### Multiplex immunoassay analysis

The levels of 256 initial analytes were measured in 250 μL serum using multiplexed immunoassays (Discovery MAP™ platform) in a Clinical Laboratory Improvement Amendments (CLIA)-certified laboratory (Myriad-RBM; Austin, TX, USA) as described previously [14]. Briefly, samples were analyzed at optimized dilutions and raw intensity measurements were converted into absolute protein concentrations using duplicate 8-point standard curves. Sample analysis was randomized to minimize bias due to measurement-related effects.

### Statistical analysis

The statistical programming software R (http://www.r-project.org/) was used to pre-process, analyze and plot the multiplex immunoassay data. First, the data were filtered to remove those assays with more than 30% of values lying outside the limits of quantitation. This resulted in exclusion of 48 assays. For the remaining 208 analytes, low values were replaced by 0.5 X the corresponding minimum values for that assay and high readings were replaced by 2.0 X the maximum levels. For each assay, values were log_e_ transformed for analysis, and outlying values were removed if these exceeded more than 3 standard deviations from the means. Deviations from typical molecular developmental patterns in ASD siblings were assessed by calculating age-diagnosis interactions. The interaction was assessed using a linear model, adjusting for additional covariates of family membership, plate, BMI, and sex. A similar procedure was used to identify molecules changed in ASD, adjusting for these same additional covariates in a linear model. Next, relationships between molecules with significant age-diagnosis interactions were tested by computing Spearman rank correlation coefficient between each pair of molecules for control siblings using untransformed data. Statistical tests were deemed significant at *P* <0.05.

### In-silico pathway analysis

The UniProt accession codes of proteins which showed diagnosis-age interactions were uploaded into the Ingenuity Pathways Knowledge Database (IPKB; Ingenuity™ Systems; Mountain View, CA, USA). Pathways most significant to the data set were determined by overlaying the identified proteins onto predefined pathway maps in the IPKB. A right-tailed Fisher’s exact test was used to calculate *P* values associated with the identified pathways. The significance of the association between the dataset and canonical pathways was measured by the ratio of the number of significant molecules divided by the total number of molecules in the canonical pathway and by the Fisher’s exact test *P* value.

## Results

### Identification of altered molecules in autism spectrum disorder individuals compared to sibling controls

Multiplex immunoassay analysis of all ASD individuals (n = 37) and controls (n = 37) resulted in identification of nine proteins which were present at significantly different levels (interleukin-3, interleukin 12 subunit p40, interleukin-13, macrophage derived chemokine, stem cell factor, Tamm-Horsfall urinary glycoprotein, tumor necrosis factor beta, tyrosine kinase with Ig and EGF homology domains 2 and von Willebrand factor) (Table 1). None showed a difference higher than 1.2-fold or less than 0.8-fold. We next determined whether molecular differences between ASD and control individuals were potentially obscured by the age range investigated.

**Table 1:** Multiplex immunoassay analysis identification of proteins significantly altered in autism spectrum disorders (ASD)

**Analytes**	***P *value**	**Fold change**^**a**^
Tamm-Horsfall urinary glycoprotein	0.004	1.18
Interleukin-3	0.010	0.79
von Willebrand factor	0.023	1.16
Interleukin 12 subunit p40	0.025	0.87
Tyrosine kinase with Ig and EGF homology domains 2	0.033	1.09
Tumor necrosis factor beta	0.034	1.18
Interleukin-13	0.038	0.86
Macrophage derived chemokine	0.042	0.92
Stem cell factor	0.050	0.89

^a^Geometric means were used to calculate fold changes (ASD/control) in ASD individuals (n = 37) compared to control (n = 37) subjects. There were no significant differences in age (ASD = 10.9 ± 3.4 years; control = 10.3 ± 3.0 years) or body mass index (BMI) (ASD = 18.0 ± 3.5 kg/m^2^, control = 17.7 ± 3.1 kg/m^2^). Autism Spectrum Quotient (AQ) scores were significantly different (ASD = 92.0 ± 20; control = 43.9 ± 18; *P* <0.001). See Table 2 for more detailed demographics. Demographics are reported as mean ± standard deviation.

### Identification of molecules which showed diagnosis-age interactions

**Figure 1:**
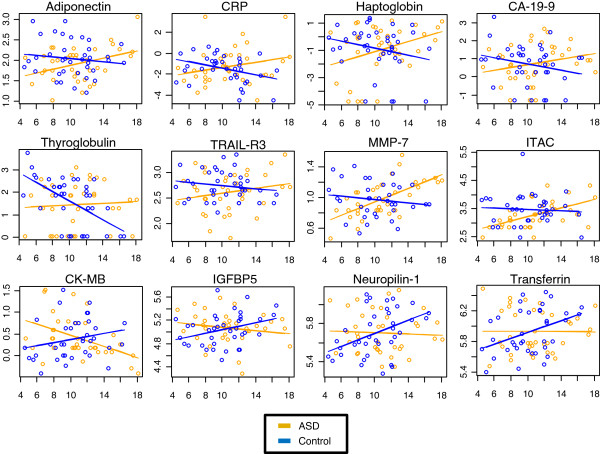
**Age-dependent changes in expression of serum proteins in 4- to 18-year-old autism spectrum disorder (ASD) subjects compared to matched sibling controls.** Protein concentrations were plotted against age after log_e_ transformation, and a linear regression was fit in ASD subjects (orange) and sibling controls (blue). The abbreviations are as described in Table 3.

The investigated individuals were separated into age groups approximating time periods before (<9 years), during (9 to 13 years) and after (>13 years) puberty (Table 2). ASD subjects and their unaffected control siblings did not differ significantly in mean age, body mass index (BMI), height or weight values. AQ scores were significantly different (*P* <0.05) between ASD and unaffected individuals. AQ scores did not change significantly with age for ASD individuals or for controls. Deviations from typical molecular developmental patterns in ASD subjects were assessed by calculating an age-diagnosis interaction using a linear model, as described in the Materials and Methods section. After adjusting for additional covariates of family membership, assay plate, BMI, and sex, 12 proteins showed significant diagnosis-age interactions (Table 3; Figure 1). None of these proteins overlapped with molecules found to be significantly different in the comparison of all ASD and control subjects (Table 2). The most significant divergences in trajectories were observed for matrix metalloproteinase 7 (MMP-7) (*P* = 0.005; increasing slope), adiponectin (*P* = 0.007; increasing slope) and transferrin (*P* = 0.012; decreasing slope). The most profound ratiometric differences across age groups were seen for haptoglobin, cancer antigen 19–9 (CA-19-9), thyroglobulin (TG) and C-reactive protein (CRP), which were present at approximately 50% of control levels in the youngest age group (<9 years) and were increased by more than 200% compared to controls in the highest age group (>13 years). Four molecules (insulin-like growth factor binding protein 5 (IGFBP5), transferrin, neuropilin-1, creatine kinase-MB (CK-MB)) showed the opposite trajectory with respect to typical molecular levels, with higher levels seen in the youngest group and lower levels in the oldest group.

**Table 2:** Demographic information after separation of autism spectrum disorder (ASD) and control subjects according to age range

	**<9 years**		**9 to 13 years**		**>13 years**	
**Diagnosis**	**ASD**	**Control**	**ASD**	**Control**	**ASD**	**Control**
N (M/F)	12 (11/1)	11 (9/2)	17 (14/3)	22 (20/2)	8(8/0)	4 (4/0)
AD/PDD-NOS	7/5	NA	11/6	NA	3/5	NA
Age	7.2 ± 1.2^a^	6.8 ± 1.6	11.2 ± 1.0	11.0 ± 1.3	15.8 ± 1.7	15.4 ± 1.0
BMI	15.9 ± 1.3	16.1 ± 1.7	17.6 ± 2.1	17.5 ± 2.4	22.2 ± 4.6	23.0 ± 4.0
Height	128 ± 10	127 ± 9	154 ± 7	148 ± 11	173 ± 9	178 ± 8
Weight	26 ± 5	26 ± 6	42 ± 8	39 ± 10	67 ± 19	73 ± 12
AQ (Total)	95 ± 16	44 ± 23	91 ± 22	46 ± 17	90 ± 21	33 ± 11

^a^Demographic variables are listed as mean ± standard deviation. *AD* autistic disorder, *AQ* Autism Spectrum Quotient, *BMI* body mass index, *PDD-NOS* pervasive developmental disorder-not otherwise specified.

**Table 3:** Multiplex immunoassay analysis identification of proteins with significant age-diagnosis interactions

**Analytes**	**Interaction *P *value**	**Fold change**^**a**^		
		**<9 years**	**9 to 13 years**	**>13 years**
Haptoglobin (HP)	0.021	0.36	1.20	2.83
Cancer antigen 19–9 (CA-19-9)	0.046	0.59	1.17	2.45
Thyroglobulin (TG)	0.021	0.32	1.35	2.39
C-Reactive Protein (CRP)	0.014	0.51	1.90	2.35
TRAIL-R3 (TR3)	0.034	0.86	0.81	1.31
Adiponectin (ADIP)	0.007	0.80	0.81	1.25
Matrix metalloproteinase 7 (MMP-7)	0.005	0.82	1.07	1.23
Interferon inducible T cell α chemoattractant (ITAC)	0.035	0.66	0.86	1.21
Insulin-like growth factor binding protein 5 (IGFBP5)	0.027	1.23	0.95	0.87
Transferrin (TF)	0.012	1.28	0.89	0.86
Neuropilin-1 (NP1)	0.020	1.24	0.92	0.83
Creatine kinase-MB (CK-MB)	0.022	1.50	0.94	0.83

^a^The data obtained for all subjects were partitioned into the indicated age bins and geometric means were used to calculate fold changes (autism spectrum disorder/control).

### Correlations of molecules with significant diagnosis-age interactions

**Figure 2:**
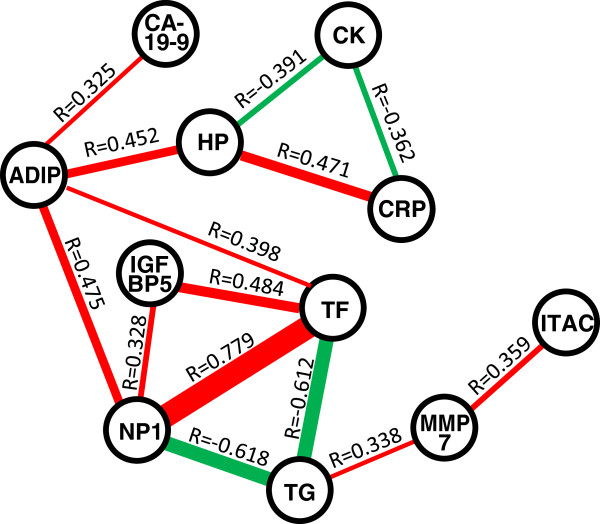
**Diagram showing significant correlations of protein levels.** The coefficient (R) is given above each connection and the width of the connections are inversely proportional to the *P* value. Red and green connections denote positive and negative correlations, respectively. The abbreviations are as described in Table 3.

Spearman rank correlation testing showed that the levels of 11 out of the 12 molecules with significant age-diagnosis interactions were also significantly correlated with at least one other molecule (Figure 2). TRAIL-R3 was the only protein that was not correlated with at least one other. Neuropilin 1 had the highest Spearman correlation coefficient and most significant correlations with the proteins transferrin (R = 0.779, *P* = 1.36E-08) and thyroglobulin (R = −0.618, *P* = 4.62E-05). Also, adiponectin, transferrin and neuropilin 1 showed the greatest number of connections by having significant correlations with four other proteins.

### Pathway analysis

The UniProt accession codes of all 12 proteins were uploaded into the Ingenuity Pathways Knowledge Base (IPKB; http://www.ingenuity.com) to identify the most over-represented pathways associated with the dataset (Table 4). This showed that the diseases most significantly associated with these proteins were hematological diseases (*P* <0.001) and endocrine system disorders (*P* <0.001). Both of these categories were linked to changes in adiponectin, creatine kinase-MB, C-reactive protein, haptoglobin, matrix metalloproteinase 7 and transferrin, although interferon inducible T cell α-chemoattractant (ITAC) was associated specifically with hematological disease and thyroglobulin was specifically related to endocrine system disorders. The most significant canonical pathway associated with the proteins was acute phase response signaling (*P* <0.001), based on changes in C-reactive protein, haptoglobin and transferrin.

**Table 4:** ***In-silico *pathway analysis of proteins with significant diagnosis-age interactions**

**Top diseases**^**a**^	***P *value**	**Molecules**
Hematological disease	1.60E-05 to 1.64E-02	ADIP, CK-MB, CRP, HP, ITAC, MMP-7, TF
Endocrine system disorders	9.51E-05 to 3.71E-02	ADIP, CK-MB, CRP, HP, MMP-7, TF, TG
**Top canonical pathway**	***P *value**	**Ratio**
Acute phase response	2.56E-04	3/172

^a^UniProt accession codes of all 12 proteins with significant diagnosis-age interactions were uploaded into Ingenuity Pathways Knowledge Base (IPKB), and the most over-represented diseases and canonical pathways were determined as described in the Materials and Methods section. The abbreviation of protein names are as indicated in Table 3. The ratio for canonical pathways represents the number of molecules from the data set divided by the total number of molecules in that pathway.

## Discussion

This is the first proteomic profiling study aimed at identifying age-related serum biomarker changes in young ASD subjects. In addition, we used well-matched non-affected siblings, allowing us to detect changes related specifically to the manifestation of ASD as a clinical state. Using multiplex immunoassay analysis of 208 molecules we identified significantly different age-dependent trajectories in the levels of 12 proteins in ASD individuals compared to unaffected sibling controls. The most significant canonical pathway associated with the age-dependent changing proteins was acute phase response, consistent with known alterations in immunological and inflammatory functions in ASD individuals [19,20]. A literature review by Rossignol and Frye highlighted 10 studies that reported an increase in prevalence of autoimmune disorders in family members of children with ASD [21], and another study has linked perturbed immune function in young autism children to gastrointestinal disturbances [22]. In addition, changes in other proteins were consistent with previous reports related to alterations in metabolism [23] and mitochondrial function [24]. Furthermore, Adams and coworkers have comprehensively reviewed the link between autism and metabolic disturbances in young and adult autistic patients [25]. Interestingly, another study showed that treatment of autism patients with pioglitazone resulted in improvement of some symptoms, with a stronger effect in younger patients [26]. This is the first report showing that changes in these molecules occur in an age-dependent manner in ASD individuals. In addition, our findings suggest that pubertal status may be an important factor to take into consideration after identifying opposing directional changes in the oldest and youngest age groups in ASD compared to unaffected individuals.

It is likely that the significantly different trajectories in the inflammation- and metabolism-related molecules with age in ASD are linked at a fundamental level [27]. For example, C-reactive protein and haptoglobin, which both increased with age in the ASD subjects, are components of the acute phase response, although these same proteins have also been used as biomarkers for immune disorders and metabolic syndrome [28,29]. We also found increased levels of TRAIL-R3, which has been linked to inflammation by regulation of apoptotic processes in immune cells [30] and also to the loss of insulin-producing pancreatic beta cells in type 1 diabetes mellitus [31]. Likewise, we found increased levels of matrix metalloproteinase (MMP) 7 in the higher age group of ASD individuals, suggestive of an inflammatory phenotype. MMPs play a pivotal role in the pathogenesis of autoimmune and inflammatory conditions such as arthritis, atherosclerosis, pulmonary emphysema and endometriosis [32]. In addition, changes in the MMPs have been linked to metabolic diseases including type 2 diabetes mellitus [33].

We also found higher levels of adiponectin with increasing age in ASD individuals compared to a decrease with age seen in the control subjects. The finding of lower levels of adiponectin in the younger age groups of ASD patients is consistent with the findings of Shimuzu *et al*., which showed decreased levels of this protein in ASD subjects compared to controls at an average age of 12 years old [34]. Adiponectin is involved in the control of fat metabolism and insulin sensitivity. Normally, low levels of this protein have been used as a biomarker for oxidative stress, diabetes and a risk factor for metabolic syndrome [35,36]. Therefore, this finding may be in contrast with the reported higher incidence of these conditions in ASD individuals [37,38]. However, this could also be due to the fact that most previous studies have not accounted for any differences in age-related trajectories. In line with this, we also found decreased levels of insulin-like growth factor binding protein 5, which is known to be involved in cell proliferation, differentiation and apoptosis [39], in diabetes and other metabolic conditions [40]. The finding that thyroglobulin levels were increased with age in ASD individuals may have metabolic links as this protein is an essential autocrine regulator of physiological thyroid follicular function that counteracts the effects of thyroid stimulating hormone [41]. Variations in thyroglobulin are associated with susceptibility to autoimmune thyroid disease type 3, which include Graves’ disease and Hashimoto thyroiditis [42].

Other potential markers of inflammation or immune function that were increased with age included cancer antigen 19–9 (CA-19-9). Although CA-19-9 has been mainly associated with pancreatic cancer [43], it has also been used a biomarker of pancreatic tissue damage as seen in type 2 diabetes and other metabolic disorders [44]. Likewise this marker is elevated in ASD individuals who have insulin resistance [45], suggesting that the ASD individuals in this study may become more susceptible to such disorders after puberty. This is consistent with the increased prevalence of metabolic conditions in young ASD individuals compared to the general public [46]. We also found high levels of creatine kinase-MB at younger ages, consistent with the findings of a previous study in children with ASD [47]. However, we found that the levels of creatine kinase decreased with age, which suggests that progressive effects may occur in energy metabolism or related pathways in ASD. This could be linked to mitochondrial dysfunction and oxidative stress that has been associated with the etiology of autism [21].

The multiplex immunoassay profiling analysis also led to identification of decreased levels of neuropilin 1 in young ASD individuals compared to controls. The neuropilin protein family has been implicated in the embryonic development of neural and vascular systems, and regulation of many processes in adults, such as angiogenesis, the vascular system and the immune response [48]. This is in line with previous reports showing effects on both of these pathways in ASD subjects [7-10,49]. Effects on the vascular system can be reflected clinically by an abnormal blood flow. Therefore, it is interesting that neuroimaging studies have identified changes in blood flow in and between certain brain regions of individuals with ASD when tested under resting and active conditions [50,51]. It should be noted that we did not find any age-related changes in the levels of BDNF as described in previous studies [12]. However, this could be due to the fact that such changes have only been described for individuals with ASD in the 0 to 9 years age range and the present study only considered participants older than 4 years of age.

## Conclusions

One limitation of this study was the potential bias in the molecular class of the investigated molecules. This procedure was based on the commercial availability of a multiplexed immunoassay platform and did not specifically target proteins of other functional classes. Therefore, it is possible that a different selection of molecules would lead to different conclusions from those drawn in this study. Another limiting factor was the small number of clinical serum samples tested using the multiplex analysis. This was due to the rarity of such samples that could be obtained using strict standard operating procedures from both ASD individuals and matched sibling controls. In addition, the samples used in this study were obtained using matched ASD individuals and controls sampled at a single time point. It would be more accurate to repeat the study under prospective conditions in which multiple samples are taken from the same subjects over time, although this is most likely impractical and will result in a high drop-out rate. Finally, the current findings should be considered as preliminary as we did not correct *P* values from the molecular analysis studies for multiple hypothesis testing. However, there have been no previous proteomic profiling studies carried out in young autism patients that have led to identification of large effects because well-controlled studies using such well-characterized patients are rare. In conclusion, we have identified 12 serum proteins involved in inflammation and metabolic dysfunction that appear to show different trajectories in ASD individuals compared to controls. The predominant effect appeared to be an age-related increase in inflammation and metabolic dysfunction. Future research in this area should incorporate the use of follow-up data from analysis of separate cohorts to confirm these findings. The study of younger subjects in prospective studies would provide further insight into the role of these proteins in ASD and enable development of more accurate, early diagnostic tests. Also, sampling from the same individuals over time will help to determine the true age-dependency of these serum protein expression changes. Furthermore, association studies that compare the protein readings with the time course of symptoms and other read-outs, such as those from functional imaging analyses [52], will be helpful in increasing our understanding of the changes which occur in ASD at different developmental stages. We anticipate that the development and application of biomarker test panels based on the current findings will lead to earlier and more accurate diagnosis and could also lead to the development of much-needed novel therapies for individuals with these conditions.

## Abbreviations

AD: Autism disorder; ASD: Autism spectrum disorder; AQ: Autism Spectrum Quotient; BDNF: Brain-derived neurotrophic factor; BMI: Body mass index; CA-19-9: Cancer antigen 19–9; CK-MB: Creatine kinase-MB; CLIA: Clinical laboratory improvement amendments; CMO: Commissie Mensgebonden Onderzoek; CRP: C-reactive protein; EGF: Epidermal growth factor; IGFBP5: Insulin-like growth factor binding protein 5; IPKB: Ingenuity Pathways Knowledge Database; ITAC: Interferon inducible T cell α-chemoattractant; MAP: Multi-analyte profiling; mRNA: Messenger ribonucleic acid; MMP: Matrix metalloproteinase; NMR: Nuclear magnetic resonance; PDD-NOS: Pervasive developmental disorder-not otherwise specified; TG: Thyroglobulin; STARD: Standards for reporting of diagnostic accuracy; TRAIL-R3: Tumor necrosis factor receptor superfamily member 10C.

## Competing interests

PCG, HR and SB are consultants for Myriad-RBM. However, this does not interfere with policies regarding sharing of data and materials as specified by the journal.

## Authors’ contributions

JMR and PCG carried out the molecular profiling data analyses, interpreted the results, prepared the figures and tables, and wrote the manuscript. JACB and HR wrote the manuscript and carried out editing. JG, NR and BF designed the clinical studies and edited the manuscript. JKB and SB conceived the study, interpreted the results and edited the manuscript. All authors read and approved the final manuscript.
